# Lesão Miocárdica após Cirurgia Não Cardíaca – Estado da Arte

**DOI:** 10.36660/abc.20200317

**Published:** 2021-09-01

**Authors:** Antonio José Lagoeiro Jorge, Evandro Tinoco Mesquita, Wolney de Andrade Martins

**Affiliations:** 1 Universidade Federal Fluminense Niterói RJ Brasil Universidade Federal Fluminense (UFF), Niterói, RJ - Brasil; 2 UHG Centro de Ensino e Treinamento Edson de Godoy Bueno Rio de Janeiro RJ Brasil Centro de Ensino e Treinamento Edson de Godoy Bueno / UHG, Rio de Janeiro, RJ - Brasil; 3 UNIALFA Colégio Brasileiro de Executivos em Saúde CBEXs São Paulo SP Brasil UNIALFA / Colégio Brasileiro de Executivos em Saúde CBEXs, São Paulo, SP - Brasil; 4 Sociedad Interamericana de Cardiología Cidade do México México Sociedad Interamericana de Cardiología (SIAC), Cidade do México - México; 5 DASA Complexo Hospitalar de Niterói Niterói RJ Brasil DASA Complexo Hospitalar de Niterói, Niterói, RJ - Brasil

**Keywords:** Contusões Miocárdicas, Biomarcadores, Cuidados Pré-Operatórios

## Abstract

Aproximadamente 300 milhões de cirurgias não cardíacas são realizadas anualmente no mundo, e eventos cardiovasculares adversos são as principais causas de morbimortalidade no período perioperatório e pós-operatório. A lesão miocárdica após cirurgia não cardíaca (MINS, do inglês *myocardial injury after non-cardiac surgery*) é uma nova entidade clínica associada com desfechos cardiovasculares adversos. MINS é definida como uma lesão miocárdica que pode resultar em necrose secundária à isquemia, com elevação dos biomarcadores. A lesão tem importância prognóstica e ocorre em até 30 dias após a cirurgia não cardíaca. Os critérios diagnósticos para MINS são: níveis elevados de troponina durante ou em até 30 dias após a cirurgia não cardíaca, sem evidência de etiologia não isquêmica, sem que haja necessariamente sintomas isquêmicos ou achados eletrocardiográficos de isquemia. Recentemente, pacientes com maior risco para MINS têm sido identificados por variáveis clínicas e biomarcadores, bem como por protocolos de vigilância quanto ao monitoramento eletrocardiográfico e dosagem de troponina cardíaca. Pacientes idosos com doença aterosclerótica prévia necessitam medir troponina diariamente no período pós-operatório. O objetivo deste trabalho é descrever este novo problema de saúde pública, seu impacto clínico e a abordagem terapêutica contemporânea.

## Introdução

**Figura 1:**
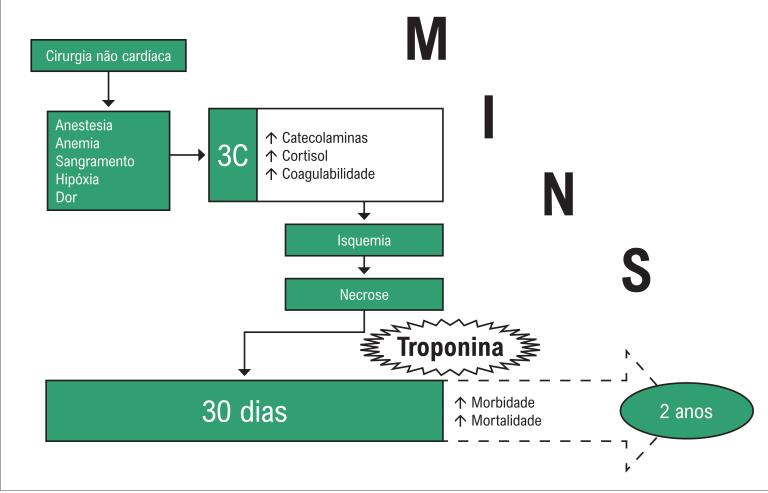
Esquema de desenvolvimento de lesão miocárdica após cirurgia não cardíaca (MINS, do inglês myocardial injury after non-cardiac surgery).

Aproximadamente 300 milhões de cirurgias não cardíacas são realizadas anualmente no mundo, e eventos cardiovasculares adversos são as principais causas de morbimortalidade no período perioperatório e pós-operatório.^1^ A lesão miocárdica após cirurgia não cardíaca (MINS, do inglês *myocardial injury after non-cardiac surgery*) é uma nova entidade clínica, diferente do infarto do miocárdio (IM) que ocorre no período pós-operatório, e associada com desfechos cardiovasculares adversos, como mostrado no registro internacional VISION.^2^ MINS é definida como uma lesão miocárdica que pode resultar em necrose secundária à isquemia, com elevação dos biomarcadores. A lesão tem importância prognóstica e ocorre em até trinta dias após a cirurgia não cardíaca.^3^ MINS tem uma incidência estimada de oito milhões de pacientes por ano, e associação independente com o risco de morte e complicações cardiovasculares no período pós-operatório inicial.^4^^,^^5^ (Figura 1).

A relevância prognóstica pode ser demonstrada pela associação dos critérios diagnósticos propostos com taxas de mortalidade de 30 dias^6^ e de dois anos.^7^

MINS não inclui lesão miocárdica perioperatória por causas não isquêmicas, tais como sepse, fibrilação atrial, embolismo pulmonar, e insuficiência renal, ou níveis de troponina cronicamente elevados.^6^^,^^7^

Os critérios diagnósticos para MINS são: níveis elevados de troponina no período de 30 dias após a cirurgia não cardíaca, considerados secundários à isquemia miocárdica, isto é, sem evidência de etiologia não isquêmica, e sem a exigência da presença de sintoma isquêmico ou achado eletrocardiográfico de isquemia.^6^

Apesar de MINS ser um fenômeno pós-operatório comum e altamente reconhecido, sua incidência, fatores de risco, fisiopatologia, e implicações clínicas não são completamente definidos.^8^ As complicações cardiovasculares são uma causa de morbidade e mortalidade em pacientes submetidos à cirurgia não cardíaca, e estudos mostraram que a incidência de IM no perioperatório corresponde a 6,2% de todas as cirurgias realizadas. A fisiopatologia dos eventos cardiovasculares no período pós-operatório é complexa, e envolve, entre outros, indução anestésica, sangramento, anemia, hipóxia, e dor no período pós-operatório, causando elevação nos níveis de catecolaminas e na síntese de cortisol, e um estado de hipercoagulabilidade.^8^ Recentemente, pacientes com maior risco para MINS têm sido identificados por variáveis clínicas e biomarcadores, bem como por protocolos de vigilância quanto ao monitoramento eletrocardiográfico e dosagem de troponina cardíaca. Assim, em pacientes idosos com doença aterosclerótica prévia, sugere-se que os níveis de troponina sejam dosados por três dias no pós-operatório.^6^

A predição de complicações cardíacas após cirurgia não cardíaca é difícil e imprecisa, e as medidas de troponina parecem exercer um papel importante nesse cenário. Estudos mostram que a troponina cardíaca ultrassensível (Tn-us) pode ser detectada na maioria dos pacientes antes da cirurgia não cardíaca, e que valores mais elevados estão associados com risco aumentado. Portanto, o uso da Tn-us pode melhorar a predição de complicações cardíacas após a cirurgia não cardíaca.^9^^,^^10^

Apesar do aumento na literatura sobre o assunto e de evidência científica, MINS é pouco reconhecido, uma vez que, atualmente, não existe consenso global sobre sua definição ou critérios diagnósticos. Muitos estudos utilizam diferentes terminologias, tais como IM no período perioperatório/pós-operatório^11^ ou lesão miocárdica perioperatória^12^ para descrever o fenômeno. Além disso, os pontos de corte para os níveis de troponina para fins diagnósticos variam entre estudos, dependendo do subtipo de troponina e do método utilizado para sua detecção (Tabela 1). Ainda, a presença de MINS tem atraído pouca atenção entre cardiologistas e médicos em hospitais, em termos do diagnóstico, implicações clínicas, e prognóstico.^13^^,^^14^ O objetivo deste artigo foi descrever esse novo problema clínico com repercussões sobre a saúde pública e a abordagem terapêutica contemporânea.

**Tabela 1:** Critérios para caracterização de lesão miocárdica, lesão miocárdica após cirurgia não cardíaca (MINS), lesão miocárdica no perioperatório, e infarto do miocárdio nos períodos perioperatório e pós-operatório

	Critérios
Lesão miocárdica^32^	Evidência de valores elevados de troponina cardíaca, com pelo menos um valor acima do limite superior de referência no percentil 99; Manifestações clínicas não precisam estar presentes; A lesão miocárdica é considerada aguda se houver elevação e/ou queda dos níveis de troponina cardíaca; Liberação de biomarcadores cardíacos sem evidência de isquemia do miocárdio;
Lesão miocárdica após cirurgia não cardíaca (MINS)^32^	Definida como lesão celular miocárdica durante os primeiros 30 dias após cirurgia não cardíaca de etiologia isquêmica e independentemente associada com mortalidade; Ausência de outras condições não isquêmicas;
Lesão miocárdica perioperatória^12^	Normalmente não exibe sintomas típicos de isquemia miocárdicas; Eletrocardiograma tem sensibilidade muito baixa para o diagnóstico; Detecção e a quantificação de lesão aguda de cardiomiócitos pela medida de troponina cardíaca; Definida como aumento absoluto nos níveis de troponina ultrassensível ≥ 14 ng/L acima dos valores pré-operatórios; Após cirurgia não cardíaca, fortemente associada com mortalidade em 30 dias
Infarto do miocárdio nos períodos perioperatório e pós-operatório^49^	Definido como elevação nos níveis de biomarcadores ou enzimas cardíacas (com definições separadas para troponina ou fração MB da creatina quinase), e um ou mais dos seguintes fatores: Sintomas isquêmicos; Alterações eletrocardiográficas em duas derivações contíguas (i.e., desenvolvimento de ondas Q patológicas, elevação do segmento ST, depressão do segmento ST, ou inversão da onda T); Intervenção da artéria coronária, ou evidência de infarto do miocárdio no exame de imagem ou necrópsia

Para estruturar este artigo de revisão, a busca de artigos foi realizada em dois bancos de dados, PubMed e Scielo, utilizando as palavras-chave em inglês “myocardial injury AND non-cardiac surgery”. A pesquisa foi realizada em janeiro de 2020. Foram incluídos estudos prospectivos e retrospectivos, e foram excluídos casos clínicos e resumos apresentados em congressos. A elegibilidade de cada estudo foi independentemente avaliada por dois investigadores. Opiniões divergentes sobre a relevância dos artigos foram resolvidas por consenso.

### Lesão miocárdica após cirurgia não cardíaca

#### Aspectos epidemiológicos

Morte cardíaca é a causa principal de mortalidade no pós-operatório nos primeiros 30 dias após cirurgia.^15^ De todas as cirurgias realizadas no mundo, estima-se que aproximadamente 100 milhões envolvem pacientes com idade igual ou superior a 45 anos; cerca de 1,1 milhão (1,1%) desses sofrem um IM com sintomas de isquemia no período perioperatório, 2,2 milhões (2,2%) tem um IM assintomático, e 4,6 milhões (4,6%) sofrem MINS. As taxas de mortalidade em 30 dias nesses três grupos são 9,7%, 12,5%, e 7,8%, o que corresponde, anualmente, a mais de 750 mil mortes por isquemia do miocárdio e, portanto, a um novo desafio de saúde pública mundial.^16^

Pacientes diagnosticados com MINS são mais velhos que aqueles sem a doença, e a incidência de MINS é significativamente mais alta em homens (17,7%) que em mulheres (16,2%).^1^ Ainda, pacientes que desenvolvem MINS têm mais fibrilação atrial, insuficiência cardíaca, doença arterial coronariana, doença renal crônica, e uma maior frequência cardíaca no pré-operatório.^17^ Mais que 90% dos pacientes com MINS não apresentam elevação do segmento ST ou qualquer outro sintoma de isquemia.^4^

Smilowitz e Berger^8^ conduziram uma meta-análise envolvendo 169 estudos, mostrando uma incidência de MINS de 17,9% das cirurgias não cardíacas. Entre os 139 estudos em que se mediram sistematicamente os biomarcadores cardíacos em todos os pacientes cirúrgicos, MINS ocorreu em 19,6% dos pacientes. Entre os outros 30 estudos, sem medidas de biomarcadores, a incidência de MINS foi de 9,9%.^1^ MINS ocorre mais frequentemente em procedimentos de urgência que em procedimentos eletivos (32,7% vs. 16,6%). A incidência também varia conforme o subtipo das cirurgias não cardíacas; a incidência de MINS foi de 10,1% e 18,0% nas cirurgias vasculares e cirurgias ortopédicas, respectivamente.

#### Fatores de risco

**Figura 2:**
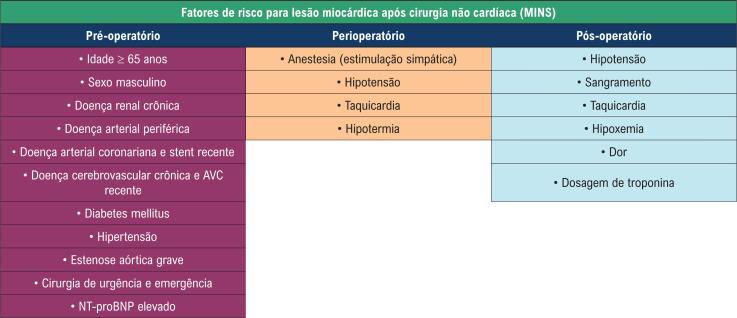
Fatores de risco nos períodos pré-operatório, perioperatório, e pós-operatório para lesão miocárdica após cirurgia não cardíaca (MINS). AVC: acidente vascular cerebral; NT-ProBNP: N-terminal do pró-hormônio do Peptídeo Natriurético do tipo B (NT-proBNP). Adaptado de Devereaux & Szczeklik W.^6^

Estudos têm relatado a prevalência de doença cardiovascular ou de pelo menos um fator de risco cardiovascular entre indivíduos submetidos a cirurgia não cardíaca.^1^ Os pacientes com MINS eram mais propensos a apresentarem pressão arterial elevada, doença arterial coronariana, IM prévio, insuficiência cardíaca, e doença renal crônica em comparação a pacientes sem MINS^1^ (Figura 2).

O risco de MINS é maior em indivíduos submetidos à cirurgia de urgência ou emergência, cirurgia aberta, transfusões, tempo de cirurgia prolongado, pressão arterial média < 65 mmHg, frequência cardíaca ≥ 110 bpm, e uso de vasopressores no período peiroperatório.^1^^,^^5^

A relação entre o tipo de anestesia e MINS continua incerta. Enquanto um estudo mostrou uma associação do sevoflurano com taxas mais baixas de MINS em comparação ao propofol (11,7% vs. 29,0%, p = 0,018),^18^ outros estudos não mostraram benefício do uso de anestésicos voláteis em relação à ocorrência de MINS.^19^^,^^20^

É importante identificar pacientes em risco elevado de eventos cardíacos adversos graves, com base na avaliação pré-operatória e baixa capacidade funcional (< 4 METs). As diretrizes recomendam avaliar os pacientes com doença cardiovascular conhecida, ou aqueles com idade igual ou superior a 45 anos que foram submetidos à cirurgia e requeiram ao menos uma noite de internação.^21^

Custos e acessibilidade são considerados determinantes importantes do método utilizado, e estudos observacionais prospectivos avaliaram a capacidade da porção N-terminal do pró-hormônio do Peptídeo Natriurético do tipo B (NT-proBNP) e do Peptídeo Natriurético tipo B (BNP) em predizer eventos cardiovasculares maiores após a cirurgia não cardíaca. Em pacientes com idade de 45 anos ou mais, com doença cardiovascular importante, ou um Índice de Risco Cardíaco Revisado (IRCR)^22^ ≥ 1, recomenda-se medir BNP ou NT-proBNP antes da cirurgia para melhorar o risco cardíaco perioperatório em vez de se realizar testes de imagens ou exame de estresse cardíaco não invasivo.^21^

Doenças pulmonares pré-existentes contribuem para a morbimortalidade perioperatória em uma intensidade similar a complicações cardíacas.^23^ As taxas de complicações são mais altas em cirurgias abdominais, torácicas, e de cabeça e pescoço devido ao impacto sobre os mecanismos respiratórios.^24^

A maioria dos pacientes submetidos à cirurgia vascular apresentam múltiplos fatores de risco e comorbidades, e há evidências crescentes de que fatores de risco cardiovasculares tradicionais também estejam associados com tromboembolismo venoso, o que aumentaria o risco de MINS. De acordo com grandes estudos prospectivos do tipo coorte, MINS influencia, em curto e em longo prazo, todas as causas de mortalidade após a cirurgia vascular.^25^

#### Fisiopatologia

A fisiopatologia do MINS ainda não foi totalmente definida. Não está clara a predominância de trombose ou de isquemia, apesar de a maioria dos casos ocorrer em pacientes com doença aterosclerótica subjacente.^4^ Dois mecanismos fisiopatológicos distintos podem ser considerados em pacientes com MINS: ruptura, fissura ou erosão da placa coronariana, com consequente trombose intraluminal que é equivalente ao infarto agudo do miocárdio do tipo 1 (IAM1), e um desequilíbrio entre o fornecimento e a demanda de oxigênio, e presença de placas instáveis, o que caracteriza o infarto agudo do miocárdio do tipo 2 (IAM 2). Estudos sugerem que ambos os mecanismos exercem importante papel na fisiopatologia do MINS no período perioperatório.^26^^-^^28^

A confirmação de MINS é feita com base nos valores elevados de troponina, segundo grandes estudos prospectivos do tipo coorte que avaliaram níveis de troponina no pós-operatório de adultos que se submeteram a cirurgias não cardíacas. MINS não inclui lesão miocárdica perioperatória secundária a uma etiologia não isquêmica documentada, incluindo fibrilação atrial paroxística, sepse, pneumonia, e embolismo pulmonar.^6^

O estudo OPTIMUS^29^ avaliou 30 pacientes que tiveram IM sem elevação do segmento ST (STEMI) após cirurgia não cardíaca, e 30 pacientes pareados que tiveram STEMI sem cirurgia. Os pacientes foram submetidos a cateterismo cardíaco e à tomografia de coerência óptica em uma média de dois dias após o diagnóstico de IM. A presença de trombo intracoronariano oclusivo como causa do IM foi observada em 13% dos casos de IM perioperatório em comparação a 67% dos casos de IM não relacionado à cirurgia (p < 0,001). As lesões culpadas apresentaram fibroateroma em 60% dos casos perioperatórios e 67% dos casos não operatórios (p = 0,52). O estudo OPTIMUS excluiu pacientes com STEMI. Estudos grandes prospectivos sugerem que STEMI corresponde de 11% a 21% dos IMs no período perioperatório.^5^^,^^30^

O estudo VISION^31^ foi um estudo prospectivo de 955 pacientes de 12 centros em oito países, que foram submetidos à angiografia coronariana por tomografia computadorizada antes da cirurgia não cardíaca. Dos 71 pacientes (7%) que apresentaram IM no período pré-operatório, em 4%, o exame mostrou artérias coronárias normais.^6^^,^^31^ Doença arterial coronariana extensa ou obstrutiva esteve presente em 72% dos pacientes que apresentaram IM no período perioperatório, e os demais 24% apresentaram no mínimo uma placa coronária com estenose menor que 50%.^6^

Estudos mostraram que um número substancial de pacientes submetidos à cirurgia não cardíaca apresenta lesões miocárdicas importante que não se enquadram na definição universal de IM. Os estudos OPTIMUS^29^ e VISION^31^ excluíram pacientes com lesão miocárdica no perioperatório que não preencheram os critérios de definição para IM.^32^ Ambos os estudos mostraram que tromboses estiveram presentes em um terço dos casos e, nos demais pacientes, o desequilíbrio entre o aporte e a demanda de oxigênio foi o possível responsável pela lesão miocárdica no perioperatório. Assim, esses dados sugerem que cerca de um terço dos casos de MINS sejam causados por trombose. Quase todos esses pacientes apresentaram estenose que predispõe os pacientes a eventos trombóticos futuros, o que facilitaria o estabelecimento de intervenções terapêuticas efetivas.^6^

MINS foi consolidado para pacientes com níveis aumentados de troponina no período pós-operatório, na ausência dos critérios para a quarta definição universal de IM.^32^ Esses pacientes podem progredir com dor torácica, e/ou alterações eletrocardiográficas típicas de IM. Ainda, foram observadas complicações cerebrovasculares e cardiovasculares em pacientes com MINS no ambiente hospitalar e fora do hospital. Os mecanismos fisiopatológicos do MINS envolvem elevações de troponina nos primeiros dias após cirurgia, e estão fortemente associados com mortalidade em 30 dias e em longo prazo. Ainda, apesar de concentrações mais altas de troponina no período pós-operatório estarem melhor associadas com complicações cardíacas, tais como IM do tipo 2, elevações pequenas estão raramente associadas com sinais evidentes de anormalidades cardíacas no período pós-operatório.^33^^,^^34^ Esses dados foram demonstrados no estudo VISION, no qual concentração de troponina de 0,02 ng/mL mostrou uma associação independente com mortalidade por causas não vasculares (RR 1,65; IC 95% 0,74–3,67).^35^ Independentemente da causa (vascular ou não vascular) de morte, MINS como resposta ao estresse da cirurgia pode ser preditora de eventos adversos.^16^

### Diagnóstico e mecanismos de MINS

A lesão miocárdica no pós-operatório não ocorre aleatoriamente, e sua ocorrência é mais provável em pacientes com doença cardiovascular. Se, por um lado, o tipo, a duração, e extensão da cirurgia são fatores que contribuem para a ocorrência de MINS, o risco basal é um determinante bem mais forte de risco de IM e mortalidade. A avaliação do risco cardíaco pré-operatório pode ajudar na escolha do paciente, dos tratamentos, tais como escolher entre procedimento endoscópico versus procedimento aberto, bem como da intensidade e duração do seguimento pós-operatório.^4^

A estimativa do risco pré-operatório geralmente utiliza uma combinação de ferramentas de avaliação de risco, testes cardíacos não invasivos e, mais recentemente e aparentemente mais promissor, o uso de biomarcadores.

Entender as mudanças fisiológicas causadas pelo estresse cirúrgico e pelo uso de anestesia é necessário para avaliar o risco perioperatório. A resposta ao estresse cirúrgico é ativada no hipotálamo pelo local da lesão tecidual, que resulta em respostas endócrina (níveis aumentados de cortisol e hormônio antidiurético), metabólica (catabolismo de carboidratos, gordura e proteínas), e inflamatória (liberação de citocinas).^24^

Os médicos geralmente utilizam informações do paciente para avaliar tolerância ao exercício como um indicador aproximado de preparo físico. No entanto, os pacientes geralmente têm pouca habilidade para estimar sua tolerância ao exercício, e talvez por isso, os médicos também tendem a subestimar a tolerância ao exercício. Contudo, mesmo estimativas ruins da tolerância ao exercício provavelmente não têm muita importância, uma vez que mesmo testes cardiopulmonares são maus preditores de risco cardiovascular pré-operatório.^36^

Estudos epidemiológicos investigaram vários critérios para o diagnóstico de MINS e demonstraram sua associação com mortalidade em 30 dias.^6^ Esses estudos estabeleceram os seguintes critérios diagnósticos para MINS: (1) ocorrência de elevação nos níveis de troponina no pós-operatório considerada como resultante de lesão isquêmica do miocárdio na ausência de outras condições não isquêmicas, tais como Tromboembolia pulmonar (TEP), sepse, miocardite, e síndrome de Takotsubo; (2) dentro de 30 dias após a cirurgia não cardíaca; (3) sem necessariamente existir sintomas ou alterações eletrocardiográficas típicas de isquemia^3^^,^^15^ (Tabela 2).

**Tabela 2:** Elevação da troponina nos primeiros dias pós-operatório e mortalidade em 30 dias

Troponina T (ng / mL)	Pacientes n (%)	Mortes 30 dias após cirurgia n (%)	HR ajustado (IC 95%)
< 0,01	13.376 (88,4)	134 (1,0)	1,0
0,02	494 (3,3)	20 (4,0)	2,41 (1,33-3,77)
0,03-0,29	1.121 (7,4)	105 (9,3)	5,00 (3,72-6,76)
≥ 0,30	142 (0,9)	24 (16,9)	10,48 (6,25-16,62)

HR: hazard ratio. Adaptado de Devereaux et al.^35^

Esses estudos também estabeleceram limiares de troponina no perioperatório para o diagnóstico de MINS: (1) troponina T > 0,03 ng/mL^11^ e (2) aumento na troponina T ultrassensível (TnT-us) de 0,02 a < 0,065 ng/mL, com um aumento absoluto de pelo menos 0,005ng/mL (o que foi independentemente associado com mortalidade de 30 dias), ou TnT-us > 0,065 ng/mL. Apesar de nenhum estudo ter estabelecidos limiares para troponina I para MINS, os médicos devem definir elevação como qualquer valor acima do limite superior de referência no percentil 99 para cada teste de troponina I.^6^

A elevação de troponina continua controversa em populações cirúrgicas específicas, tal como pacientes idosos com fraturas no quadril. Alguns estudos mostraram um aumento na mortalidade em curto e em longo prazo,^37^^,^^38^ enquanto outros não mostraram essa alteração.^39^^,^^40^ Um estudo observacional^41^ do tipo coorte nessa população incluiu 312 pacientes e mostrou que o aumento isolado de troponina não foi preditor de morte e/ou readmissão, ou de qualquer outro desfecho incluindo complicações pós-operatórias, internação, admissão na unidade de terapia intensiva (UTI).

Estudos prospectivos avaliando adultos submetidos à cirurgia não cardíaca e medidas de troponina após a cirurgia mostraram que de 13% a 18% desenvolvem MINS em 30 dias após cirurgia.^3^^,^^12^ Entre os indivíduos que desenvolvem MINS, 22% a 29% preencheram os critérios para a definição universal de IM.^32^ A maioria do IM e MINS ocorrem nas primeiras 48 horas após a cirurgia, e o IM no perioperatório pode ocorrer sem sintomas isquêmicos. Tal disparidade pode ser explicada, em parte, pelo uso de narcóticos e sedativos após a cirurgia.^6^

**Figura 3:**
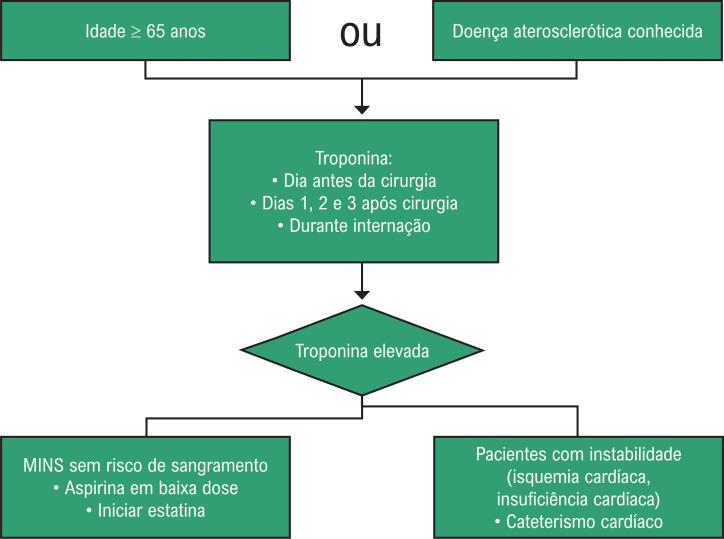
Avaliação dos pacientes submetidos à cirurgia não cardíaca Adaptado de Devereaux & Szczeklik W.^6^

Considerando que muitos casos de MINS passarão despercebidos na ausência do monitoramento de troponina, a recomendação é medir os níveis do marcador nos dias 1, 2 e 3 após cirurgia não cardíaca enquanto o paciente estiver no hospital.^6^ Com base na análise de custo-consequência do estudo VISION, podemos definir os pacientes em risco aqueles com idade superior a 65 anos ou com uma história de doença aterosclerótica.^42^ (Figura 3)

BNP e NT-proBNP são biomarcadores liberados na circulação sistêmica em resposta à distensão do miocárdio do átrio esquerdo. Também são liberados em resposta à isquemia, inflamação, e estímulos neuroendócrinos. Concentrações de peptídeos natriuréticos no pré-operatório são fortes preditores de eventos cardíacos no perioperatório, incluindo mortalidade, IM, e insuficiência cardíaca.^43^ Em pacientes submetidos à cirurgia vascular, a medida de peptídeos natriuréticos para avaliação de risco pré-operatório melhora substancialmente as predições feitas por instrumentos tais como o IRCR.^22^ Recentemente, uma subanálise do registro VISION mostrou que em indivíduos com idade maior que 45 anos, a dosagem de NT-proBNP no período pré-operatório identificou indivíduos em maior risco de MINS, e promoveu valores incrementais sobre o IRCR em identificar indivíduos em maiores riscos cardiovasculares.^44^

Rodseth et al.^45^ conduziram uma revisão sistemática de 2179 pacientes, e mostraram que níveis pré-operatórios elevados de BNP em concentrações maiores que 92 ng/L ou concentrações pré-operatórias de NT-proBNP maiores que 300 ng/L eram fortes preditores de morte e IM não fatal em 30 dias após a cirurgia (OR, 3,4 [IC 95% 2,6–4,5; p < 0,001]) e de 180 dias ou mais após cirurgia (OR 2,6 [IC 95% 2,0 –3,4; p < 0,001]). Um modelo utilizando BNP no pré-operatório reclassificou corretamente 16% mais pacientes como em alto risco, e 15% mais pacientes como em baixo risco em comparação a um modelo em que utilizou somente fatores de risco basais no pré-operatório. Com a inclusão das medidas de BNP ou NT-proBNP, observou-se que valores elevados no pré-operatório aumenta a capacidade preditiva de um desfecho composto de morte e IM não fatal em 30 dias (OR ajustado 2,2 [IC 95% 1,9– 2,7]) após cirurgia não cardíaca.^45^

Diretrizes da Sociedade Europeia de Anestesiologia para avaliação de risco operatório de cirurgia não cardíaca recomendam a medida de peptídeos natriuréticos no período pré-operatório em pacientes em alto risco para cirurgia geral ou ortopédica, e em pacientes em risco intermediário ou alto para cirurgia vascular ou torácica.^46^

As diretrizes da Sociedade Canadense de Cardiologia para cirurgias não cardíacas^21^ recomendam medir BNP ou NT-proBNP antes da cirurgia para melhorar a estimativa de risco cardíaco pré-operatório em pacientes com idade de 65 anos ou mais. Nos pacientes com idade entre 45 e 64 anos, doença cardiovascular importante e um escore de IRCR ≥ 1. Além disso, pacientes com elevada concentração de biomarcadores precisam ter os níveis de troponina medida nos primeiros dois dias após cirurgia.^21^

A Terceira Diretriz de Avaliação Cardiovascular Perioperatória da Sociedade Brasileira de Cardiologia recomenda, para pacientes em risco intermediário ou alto, a busca ativa pela ocorrência de eventos cardiovasculares, por meio de monitoramento na UTI ou na unidade de terapia semi-intensiva. O risco de um evento de natureza isquêmica requer monitoramento eletrocardiográfico e medida de marcadores de lesão miocárdica (troponina) até o terceiro dia pós-cirurgia, período em que a maioria dos eventos cardiovasculares se concentram.^47^

### Tratamento

A complexidade dos mecanismos que contribuem para a ocorrência de IM no período perioperatório torna difícil o tratamento de MINS. Algumas das opções terapêuticas estabelecidas para o cenário não operatório, tais como o uso de agentes antiplaquetários, são de alto risco devido ao risco de sangramento no pós-operatório. Além disso, existem poucos dados de ensaios clínicos randomizados sobre o tratamento de MINS.^16^

### Terapia no pré-operatório para a prevenção de MINS

Um ponto que permanece relativamente pouco abordado em MINS é a prevenção. Os mecanismos propostos de IM no período perioperatório incluem fissura ou ruptura da placa aterosclerótica, em uma condição caracterizada por hipotensão, altos níveis de catecolaminas, e arritmias. Na prevenção de MINS, deve-se estabelecer um equilíbrio entre garantir uma adequada cobertura antitrombótica sem aumentar o risco de sangramento e minimizando os efeitos adversos de um aumento no impulso simpático, evitando-se hipotensão.^14^

Até o presente momento, o cuidado perioperatório do paciente tem sido amplamente focado na prevenção de IM, e poucos estudos tentaram determinar o impacto de terapias preventivas secundárias para IM em pacientes com elevação isolada de troponina.^48^

No passado, os betabloqueadores eram recomendados a pacientes em risco vascular que se submeteram a cirurgias cardíacas de risco alto ou intermediário. Contudo, após a publicação do estudo POISE,^49^ que avaliou o uso de betabloqueadores no pré-operatório, a razão risco/benefício foi considerado desfavorável. O estudo POISE recrutou 8351 pacientes e mostrou que o metoprolol reduziu significativamente a incidência de IM em comparação ao placebo, mas aumentou significativamente o risco de acidente vascular cerebral e bradicardia clinicamente importante. Ainda, o grupo que recebeu metoprolol apresentou um risco significativamente maior de mortalidade em comparação a placebo. Assim, a principal estratégia no pré-operatório de se usar betabloqueadores mostrou-se ineficaz e mesmo perigosa para aqueles pacientes que ainda não usam esse medicamento para condições cardíacas pré-existentes.^49^ Seria apropriado que pacientes com doença arterial coronariana comprovada e aqueles com alto risco para a doença, utilizasse betabloqueadores para os benefícios dos desfechos em longo prazo, independentemente de qualquer cirurgia iminente.^50^

Dados observacionais no período perioperatório sugerem que pacientes com MINS beneficiam-se de terapia com aspirina e estatina. Apesar de o uso de aspirina ajudar a evitar o estado de hipercoagulabilidade pós-operatória que pode resultar em eventos isquêmicos, o papel da aspirina na prevenção de MINS permanece controverso.^10^ Em um subestudo com 415 pacientes que tiveram IM perioperatório no estudo POISE, uma análise multivariada demonstrou um baixo risco de morte em 30 dias em pacientes usando aspirina e estatinas.^6^ Por outro lado, os resultados do estudo POISE 2 mostraram que a aspirina não resulta em melhora na taxa de eventos cardiovasculares ou mortalidade em 30 dias, causando um aumento significativo na ocorrência de sangramento em comparação a placebo. Os autores postularam que o aumento nos eventos cardiovasculares pode estar relacionado a aumento de sangramento, causando uma falha na relação fornecimento e demanda.^51^

No entanto, o estudo POISE 2^51^ apresenta falhas que potencialmente limitam sua aplicabilidade. Por exemplo, o tempo de administração de aspirina no período pré-operatório não foi padronizado. Com base nos critérios de inclusão, a aspirina teria sido indicada para prevenção secundária na maioria dos pacientes do estudo. Retirar a aspirina desses pacientes os colocaria em um risco aumento para eventos cardíacos e acidente vascular cerebral, uma vez que a descontinuidade do tratamento com aspirina resulta em aumento nos níveis de tromboxano A2 e fibrinólise diminuída.^52^

Um estudo observacional de Foucrier et al.^53^ incluiu 66 pacientes que tiveram aumento na troponina após cirurgia vascular e 132 controles pareados que não tiveram elevação de troponina após a cirurgia. O desfecho primário foi ocorrência de desfecho cardíaco maior – IM, revascularização coronária, e edema pulmonar com hospitalização em um ano. A intensificação da medicação cardiovascular foi definida como a introdução ou aumento da dose de pelo menos um dos quatro medicamentos – agentes antiplaquetários, estatinas, betabloqueadores, e inibidores de enzima conversora de angiotensina. Pacientes sem tratamento cardiovascular intensificado apresentaram um RR de 1,77 (IC 95% de 1,13 – 2,42) para o desfecho primário, em comparação ao grupo controle. Por outro lado, os pacientes com tratamento intensificado apresentaram um risco similar para o desfecho primário em comparação ao grupo controle (RR 0,63; IC 95% 0,10-1,19).^53^ Ainda, uma meta-análise mostrou que a interrupção do uso de aspirina no período perioperatório em pacientes com ou sem risco de doença cardíaca isquêmica foi associada com um risco três vezes maior de eventos adversos cardíacos sérios.^54^

Complicações cardiovasculares são a causa mais importante de morbidade e mortalidade perioperatória em pacientes submetidos à cirurgia vascular. Os efeitos protetores relacionados ao uso de estatinas parecem ser baseados na ação hipolipemiante e outras propriedades desses medicamentos, tais como melhora na função endotelial, hemostasia, e inflamação, que resultam na estabilização da placa coronária. Ensaios clínicos mostraram que a incidência de eventos cardiovasculares nos primeiros seis meses após a cirurgia, incluindo morte por causas cardíacas, IM agudo não fatal, acidente vascular cerebral isquêmico, e angina instável, pode diminuir com o uso de atorvastatina no perioperatório em pacientes que precisam ser submetidos à cirurgia vascular, independentemente das concentrações séricas de colesterol.^55^

A terceira Diretriz de Avaliação Cardiovascular Perioperatória da Sociedade Brasileira de Cardiologia recomenda que, para pacientes recebendo aspirina para prevenção secundária, a medicação deve ser mantida em dose máxima de 100 mg por dia. Dados de meta-análise sugerem que essa relação é favorável para a maioria dos pacientes no período perioperatório. Neurocirurgias, devido à alta morbidade e mortalidade dos sangramentos relacionados, são uma indicação de suspensão do uso de aspirina sete dias antes da cirurgia.^47^

Todas as principais diretrizes recomendam a continuidade da terapia com estatina no período perioperatório, e o início do uso de estatinas em pacientes submetidos à cirurgia vascular.^21^^,^^47^^,^^56^^,^^57^ O estudo VISION mostrou que pacientes que tomaram estatinas no período pré-operatório apresentaram taxas significativamente mais baixas de MINS e de mortalidade por todas as causas. As estatinas devem ser iniciadas pelo menos duas semanas antes da cirurgia para promover os efeitos de estabilização de placa e anti-inflamatórios.^14^

As últimas diretrizes do *American College of Cardiology*/*American Heart Association* (ACC/AHA) não recomendam a revascularização coronária antes da cirurgia não cardíaca.^57^ A revascularização da artéria coronária no pré-operatório não reduziu a mortalidade em longo prazo ou o IM no período pós-operatório em comparação ao uso de medicamentos em um estudo com mais de 5000 pacientes submetidos à cirurgia vascular.^58^ A cirurgia de revascularização do miocárdio e a intervenção coronária percutânea são procedimentos associados com um risco significativo.^59^ Pacientes com *stents* coronários após a intervenção coronária percutânea estão em risco de trombose de *stent* durante a cirurgia, especialmente em caso de interrupção no uso de agentes antiplaquetários no período perioperatório. A revascularização antes da cirurgia não cardíaca é recomendada apenas para pacientes a quem a revascularização é indicada independentemente de cirurgia.^57^

Na fase perioperatória, para prevenir eventos cardiovasculares, o anestesiologista deve limitar a hipotermia e imediatamente tratar hipotensão e taquicardia, possivelmente evitando o uso de betabloqueadores.^48^

### Cuidado no pós-operatório

No período pós-operatório, hipotensão, taquicardia, hipóxia, sangramento e dor promovem um desequilíbrio entre fornecimento e consumo de oxigênio no miocárdio, aumentando o risco de lesão, devendo ser detectado e corrigido precocemente.^48^

No manejo de MINS, o uso de aspirina e estatina pode ser recomendado em pacientes que não preenchem os critérios de síndrome coronária aguda, uma vez que tal medida mostrou melhorar os resultados.^21^ Teoricamente, os betabloqueadores podem contribuir no manejo do desequilíbrio entre o fornecimento e a demanda de oxigênio que resulta em MINS. No entanto, deve-se pesar os benefícios de se usar betabloqueadores e bloqueadores de receptores da angiotensina contra o risco de hipotensão, o que pode acentuar o dano no miocárdio.^14^ Outros estudos são necessários para se obter evidências mais robustas acerca do uso desses medicamentos. Pacientes que preenchem os critérios para síndrome coronária aguda devem ser tratados segundo diretrizes clínicas atuais.^14^ Procedimentos invasivos para investigar doença coronária obstrutiva em pacientes com MINS não são indicados, exceto para pacientes com critérios para STEMI, ou pacientes com instabilidade hemodinâmica ou elétrica.

O estudo MANAGE avaliou o uso de dabigatrana para o tratamento de MINS. Os autores encontraram que os indivíduos no braço da intervenção apresentaram taxas mais baixas de eventos vasculares maiores (n = 97; 11% - pacientes alocados para dabigatrana vs. n = 133; 15% - pacientes alocados no grupo controle, p = 0,0115), com complicações hemorrágicas em comparação ao braço do placebo n = 29; 3% - pacientes alocados para dabigatrana vs. n = 31; 4% - pacientes alocados no grupo controle, p = 0,76).^7^ No entanto, o estudo tem algumas limitações no delineamento e nos resultados. O estudo foi finalizado precocemente, o desfecho primário modificado no meio do estudo, a as taxas de descontinuação do medicamento foram altas. Apesar de suas deficiências, o estudo abre caminho para mais pesquisa na área.^14^

## Conclusões

MINS representa uma nova entidade clínica caracterizada hoje como um problema de saúde emergente em vista do número crescente de cirurgias não cardíacas e sua alta prevalência. MINS é um marcador de eventos vasculares futuros, e sua detecção precoce com o uso de biomarcadores deve ser realizada em todas as cirurgias médias e grandes realizadas em indivíduo com 65 anos de idade ou mais, e naqueles com doença aterosclerótica prévia independentemente da idade. Registro multicêntricos e ensaios clínicos têm sido desenvolvidos com a ajuda da medicina perioperatória, com o objetivo de melhorar a vigilância cardiovascular e o tratamento desse grupo de pacientes.
