# Supplementary material for: Lesão Miocárdica após Cirurgia Não Cardíaca – Estado da Arte
Source: Arq Bras Cardiol. 2021 Sep 1;117(3):544–53. [Article in Portuguese] doi: 10.36660/abc.20200317 (PMC8462967; doi:10.36660/abc.20200317)
Supplement: Translation

# Translation: Myocardial Injury after Non-cardiac Surgery – State of the Art

**Authors:** Antonio José Lagoeiro Jorge, Evandro Tinoco Mesquita, Wolney de Andrade Martins

**Affiliations:** 1 Niterói RJ Brazil Universidade Federal Fluminense (UFF), Niterói, RJ - Brazil; 2 Rio de Janeiro RJ Brazil Centro de Ensino e Treinamento Edson de Godoy Bueno / UHG, Rio de Janeiro, RJ - Brazil; 3 São Paulo SP Brazil UNIALFA / Colégio Brazileiro de Executivos em Saúde CBEXs, São Paulo, SP - Brazil; 4 Cidade do México México Sociedad Interamericana de Cardiología (SIAC), Cidade do México - México; 5 Niterói RJ Brazil DASA Complexo Hospitalar de Niterói, Niterói, RJ - Brazil

**Keywords:** Myocardial Contusions, Biomarkers, Preoperative Care

## Abstract

Approximately 300 million non-cardiac surgeries are performed annually worldwide and adverse cardiovascular events are the main cause of morbidity and mortality in the peri- and postoperative period. Myocardial injury after non-cardiac surgery (MINS) is a new clinical entity associated with adverse cardiovascular outcomes. MINS is defined as myocardial injury that can result in necrosis due to ischemia, marked by increase in biomarker levels. It has prognostic relevance and occurs within up to 30 days after non-cardiac surgery. The diagnostic criteria for MINS are an elevated postoperative measure of troponin judged as secondary to myocardial ischemia, i.e., with no evidence of a non-ischemic etiology, during or within 30 days after non-cardiac surgery, and without the requirement of an ischemic symptom or electrocardiographic finding of ischemia. Recently, patients at higher risk for MINS have been recognized using clinical variables and biomarkers and established protocols for greater surveillance in relation to electrocardiographic monitoring and cardiac troponin dosage. Elderly patients with previous atherosclerotic disease need to measure troponin daily in the postoperative period. The aim of the present work is to describe this new public health problem, its clinical impact and contemporary therapeutic approach.

## Introduction

Approximately 300 million non-cardiac surgeries are performed annually in the world and adverse cardiovascular events are the main cause of morbidity and mortality in the peri- and postoperative period.^1^ Myocardial injury after non-cardiac surgery (MINS) is a new clinical entity, distinct from myocardial infarction (AMI) thath occurs in the postoperative period, associated with adverse cardiovascular outcomes as shown in the international VISION registry.^2^ MINS is defined as myocardial injury that can result in necrosis secondary to ischemia, with elevation of biomarkers. It has prognostic relevance and occurs in the period of up to thirty days after non-cardiac surgery.^3^ MINS has an estimated incidence of eight million patients per year and is independently associated with the risk of death and cardiovascular complications in the initial postoperative period^4^^,^^5^ (Figure 1).

**Figure 1:** Scheme of the development of myocardial injury after non-cardiac surgery (MINS).

The prognostic relevance can be demonstrated by the association of the proposed diagnostic criteria with the risk of mortality at 30 days^6^ and up to two years after non-cardiac surgery.^7^

MINS does not include perioperative myocardial injury due to non-ischemic causes, such as sepsis, atrial fibrillation, pulmonary embolism, and renal failure, or chronically elevated troponin levels.^6^^,^^7^

The diagnostic criteria for MINS are high troponin levels within 30 days after non-cardiac surgery judged as secondary to myocardial ischemia, i.e., with no evidence of a non-ischemic etiology, and without the requirement of an ischemic symptom or ischemia electrocardiographic finding.^6^

Although MINS is a common and increasingly recognized postoperative phenomenon, its incidence, risk factors, pathophysiology and clinical implications are not yet fully defined.^8^ Cardiovascular complications are a cause of morbidity and mortality in patients undergoing non-cardiac surgery and studies have shown that the incidence of perioperative AMI corresponds to 6.2% of all surgeries performed. The pathophysiology of cardiovascular events in the postoperative period is complex and involves, among others, anesthetic induction, bleeding, anemia, hypoxia and pain in the postoperative period, causing elevation of catecholamines and cortisol production and a state of hypercoagulability.^8^ Recently, patients at higher risk for MINS have been identified by clinical variables and biomarkers, as well by surveillance protocols in relation to electrocardiographic monitoring and cardiac troponin dosage. Thus, it is suggested that elderly patients with previous atherosclerotic disease need to measure troponin for three days postoperatively.^6^

Predicting cardiac complications after non-cardiac surgery is difficult and inaccurate, and troponin measurements seem to play an important role in this scenario. Studies have shown that high-sensitivity troponin (Hs-cTn) can be detected in most adult patients before non-cardiac surgery and that higher values are associated with increased risk. Therefore, the use of Hs-cTn can improve the prediction of cardiac complications after non-cardiac surgery.^9^^,^^10^

Despite the increase in the literature on the subject and scientific evidence, MINS is poorly recognized, as there is currently no global consensus on its definition or diagnostic criteria. Many studies have used different terminologies, such as POMI^11^ (perioperative myocardial infarction/postoperative period) or PMI^12^ (perioperative myocardial injury) to describe the phenomenon. In addition, the cutoff points of troponin levels for diagnostic purposes have varied among studies, depending on the troponin subtype and the assay used (Table 1). Also, the presence of MINS has drawn little attention among cardiologists and hospitalists in clinical practice, regarding its diagnosis, clinical implications and prognosis.^13^^,^^14^ The aim of this article is to describe this new clinical problem with repercussions on public health and the contemporary therapeutic approach.

**Table 1:** Criteria for the characterization of myocardial injury, myocardial injury after noncardiac surgery (MINS), perioperative myocardial injury, and perioperative/postoperative myocardial infarction

	Criteria
Myocardial Injury^32^	Evidence of elevated cardiac troponin values with at least one value above the 99^th^ percentile upper reference limit; Clinical manifestations do not have to be present; The myocardial injury is considered acute if there is a rise and/or fall of cardiac troponin values; Cardiac biomarker release without evidence of myocardial ischemia;
myocardial injury after noncardiac surgery (MINS)^32^	Defined as myocardial cell injury during the first 30 days after noncardiac with ischemic etiology and is independently associated with mortality; Absence of other non-ischemic conditions;
Perioperative myocardial injury^(^^12^^)^	Commonly does not exhibit typical symptoms of myocardial ischemia; Electrocardiography has very low sensitivity for diagnosis; Detection and quantification of acute cardiomyocyte injury by measuring cardiac troponin; Defined as an absolute increase in high-sensitivity troponin of ≥14 ng/L above preoperative values; After noncardiac surgery, strongly associated with 30-day mortality
Perioperative/ postoperative myocardial infarction^49^	Defined as an elevated cardiac biomarker or enzyme levels (with separate definitions for troponin or creatine kinase MB fraction), and one or more of the following: Ischemic symptoms; Electrocardiographic changes in two contiguous leads (i.e., development of pathologic Q waves, ST-segment elevation, ST-segment depression, or T-wave inversion); coronary artery intervention, or evidence of AMI on cardiac imaging or autopsy;

To structure this review article, two databases (Pubmed and Scielo) were searched, using the keywords in English “myocardial injury AND non-cardiac surgery”. The survey took place in January 2020. Prospective and retrospective studies were included, and clinical cases and abstracts presented at conferences were excluded. The eligibility of each study was independently assessed by two investigators. Divergent opinions regarding the relevance of the articles were resolved by consensus.

### Myocardial injury after non-cardiac surgery

#### Epidemiological aspects

Cardiac death is the leading cause of postoperative mortality in the first 30 days after surgery.^15^ It is estimated that among all surgeries performed in the world, approximately 100 million involve patients aged 45 years and over, nearly 1.1 million (1.1%) of these have a AMI with ischemic symptoms in the perioperative period, 2.2 million (2.2%) have an asymptomatic AMI, and 4.6 million (4.6%) have MINS. Thirty-day mortality rates in these three groups are 9.7%, 12.5% and 7.8%, respectively, which corresponds to more than 750 thousand deaths due to myocardial ischemia annually and thus a new global public health challenge.^16^

Patients diagnosed with MINS are older than those without the disease and the incidence of MINS is slightly higher in men (17.7%) than in women (16.2%).^1^ Also, patients who develop MINS have more atrial fibrillation, heart failure, coronary artery disease, chronic kidney disease and have a higher preoperative heart rate.^17^ More than 90% of patients with MINS do not present elevation of the ST segment or any other symptom of ischemia.^4^

Smilowitz & Berger^8^ carried out a meta-analysis involving 169 studies, showing that the incidence of MINS was 17.9% of non-cardiac surgeries. Among the 139 studies that systematically measured cardiac biomarkers in all surgical patients, MINS occurred in 19.6% of patients. Among the other 30 studies, without biomarker measurements, the incidence of MINS was 9.9%.^1^ MINS occurs more frequently in urgent procedures than in elective procedures (32.7% *vs*. 16.6%). The incidence also varies according to the subtype of non-cardiac surgery; the incidence of MINS was 20.1% and 18.0% in vascular surgeries and orthopedic surgeries, respectively.

#### Risk factors

Studies have reported the prevalence of cardiovascular disease or at least one cardiovascular risk factor among individuals undergoing non-cardiac surgery.^1^ Patients with MINS were more likely to have high blood pressure, coronary artery disease, previous MI, heart failure, and chronic kidney disease compared to patients without MINS^1^ (Figure 2).

**Figure 2:** Risk factors for myocardial injury in non-cardiac surgery in the pre-, peri- and postoperative periods; NT-proBNP: N-terminal prohormone of brain natriuretic peptide. Adapted from Devereaux & Szczeklik W.^6^

The risk of MINS is higher in individuals undergoing urgent or emergency surgery, open surgery, transfusions, prolonged surgery time, mean arterial pressure < 65 mmHg, heart rate ≥ 110 bpm and use of vasopressors in the perioperative period.^1^^,^^5^

The relationship between the type of anesthesia and MINS remains uncertain. While sevoflurane has been associated with lower MINS rates than propofol (11.7% *vs*. 29.0%, p = 0.018) in one study,^18^ other studies have shown no benefit from volatile anesthetics in relation to the occurrence of MINS.^19^^,^^20^

It is important to identify patients at high risk of serious adverse cardiac events based on preoperative assessment and low functional capacity (< 4 METs). Guidelines recommend evaluating those patients with known cardiovascular disease or aged 45 years or older who have undergone surgery that requires at least one night of hospitalization.^21^

Cost and accessibility are considered important determinants of the method used, and prospective observational studies have assessed the prognostic capability of the N-terminal prohormone of brain natriuretic peptide (NT-proBNP) and brain natriuretic peptide (BNP) to predict major cardiovascular events after non-cardiac surgery. In patients aged 45 years or older with significant cardiovascular disease or with a Revised Cardiac Risk Index (RCRI) score ^22^ ≥ 1 it is recommended to measure BNP or NT-proBNP before surgery to improve the estimate perioperative cardiac risk instead of imaging and noninvasive cardiac stress testing.^21^

Pre-existing lung diseases contribute to perioperative morbidity and mortality at a similar intensity to cardiac complications.^23^ Complication rates are higher in abdominal, thoracic, and head and neck surgeries due to the impact on respiratory mechanisms.^24^

Most patients undergoing vascular surgery have multiple risk factors and comorbidities and there is growing evidence that traditional cardiovascular risk factors are also associated with venous thromboembolism, which would increase the risk of MINS. According to large prospective cohort studies, MINS influences in the short and long term all causes of mortality after vascular surgery.^25^

#### Pathophysiology

The pathophysiology of MINS is not yet fully defined. It is not clear about the predominance of thrombosis or ischemia, although most cases occur in patients with underlying atherosclerotic disease.^4^ Two distinct pathophysiological mechanisms can be considered in patients with MINS: rupture, fissure, or erosion of the coronary plaque with consequent intraluminal thrombosis that is equivalent to type 1 acute MI (type 1 AMI) and an imbalance between myocardial oxygen supply and/or demand and presence of unstable plaques that characterizes type 2 acute MI (type 2 AMI). Studies suggest that both mechanisms play an important role in the pathophysiology of MINS in the perioperative period.^26^^-^^28^

MINS confirmation is made based on elevated troponin levels, according to large prospective cohort studies that evaluated post-operative troponin levels in adults undergoing non-cardiac surgery. MINS does not include perioperative myocardial injury secondary to a documented non-ischemic etiology, including paroxysmal atrial fibrillation, sepsis, pneumonia, and pulmonary embolism.^6^

The OPTIMUS^29^ study evaluated 30 patients who had a MI without ST elevation (STEMI) after non-cardiac surgery and 30 paired patients who had a non-operative STEMI. The study patients underwent cardiac catheterization and optical coherence tomography on average two days after the diagnosis of AMI. Intracoronary occlusive thrombus as the cause of AMI was present in 13% of cases of perioperative MI compared to 67% of cases of MI not related to surgery (p < 0.001). The culprit lesions showed fibroatheroma in 60% of the perioperative cases and 67% of the non-operative cases (p = 0.52). The OPTIMUS study excluded patients with STEMI. Large prospective studies suggest that STEMI accounts for 11% to 21% of perioperative MIs.^5^^,^^30^

The VISION study^31^ was a prospective cohort study of 955 patients from 12 centers in eight countries, who underwent coronary computed tomographic angiography (CTA) before non-cardiac surgery. Of the 71 patients (7%) who had AMI in the preoperative period, in 4% CTA showed normal coronaries.^6^^,^^31^ Obstructive or extensive coronary artery disease was present in 72% of patients who had AMI in the perioperative period, and the remaining 24% had at least one coronary plaque with stenosis less than 50%.^6^

Studies have shown that a substantial number of patients undergoing non-cardiac surgery have important myocardial injuries that will not meet the universal definition of MI. The OPTIMUS^29^ and VISION^31^ studies excluded patients who had perioperative myocardial injury that did not meet the definition criteria for MI.^32^ Both studies showed that thrombosis was present in one third of the cases and in the other patients, the imbalance between oxygen supply and demand was possibly the responsible for perioperative myocardial injury. Therefore, these data suggest that nearly one third of MINS cases are caused by thrombosis. Almost all these patients had underlying coronary stenosis that predispose patients to future thrombotic events, which would facilitate the establishment of effective therapeutic interventions.^6^

MINS has been consolidated for patients with increased troponin in the postoperative period in the absence of the criteria for the fourth universal definition of MI.^32^ These patients may progress with chest pain and/or electrocardiographic changes typical of MI. In addition, cardiovascular and cerebrovascular complications have been observed in in-hospital and out-hospital patients with MINS. The pathophysiological mechanisms of MINS involve elevations of troponin in the first days after surgery and are strongly associated with mortality at 30 days and in the long run. Also, although higher concentrations of troponin in the postoperative period are better associated with cardiac complications such as type 2 MI, low troponin elevations are rarely associated with evident signs of cardiac abnormalities in the postoperative period.^33^^,^^34^ These data were demonstrated in the VISION study, in which a troponin of 0.02ng/mL was independently associated with mortality from non-vascular causes (RR 3.25, 95% CI 1.78-5.94), but not with vascular causes (RR 1.65, 95% CI 0.74–3.67).^35^ Regardless of whether patients have a vascular or non-vascular death, MINS as a response to the stress of surgery may be a predictor of adverse events.^16^

### MINS diagnosis and mechanisms

Postoperative myocardial injury does not occur at random and is more likely in cardiovascular disease patients. If, on the one hand, the type, duration, and extent of surgery are factors that contribute to the occurrence of MINS, the baseline risk is a much stronger determinant of MI risk and mortality. The assessment of preoperative cardiac risk can help guide patient selection, treatment decisions such as, open *versus* endoscopic procedure and the intensity and duration of postoperative follow-up.^4^

Preoperative risk estimation generally uses a combination of risk assessment tools, non-invasive cardiac tests and, more recently and apparently more promising, the use of biomarkers.

Understanding the physiological changes due to surgical stress and the use of anesthesia is necessary to assess perioperative risk. The response to surgical stress is activated in the hypothalamus by the site of tissue damage, which results in endocrine (increased levels of cortisol and antidiuretic hormone), metabolic (catabolism of carbohydrates, fat, and proteins) and inflammatory (release of cytokines) responses.^24^

Physicians often use patient information to assess exercise tolerance as an approximate index of physical fitness. However, patients generally have little ability to estimate their tolerance to exercise, and perhaps that is why physicians also underestimate exercise tolerance. However, poor estimates of exercise tolerance probably do not matter much, because even the cardiopulmonary test poorly predicts preoperative cardiovascular risk.^36^

Epidemiological studies have investigated several criteria for the diagnosis of MINS and demonstrated their association with 30-day mortality.^6^ These studies established the following diagnostic criteria for MINS: (1) elevated postoperative troponin levels considered as a result of myocardial ischemic injury in the absence of other non-ischemic conditions such as Pulmonary Tromboembolism (PTE), sepsis, myocarditis, Takotsubo cardiomyopathy; (2) within thirty days after non-cardiac surgery; (3) and without the requirement of symptoms or electrocardiographic changes of ischemia^3^^,^^15^ (Table 2).

**Table 2:** Elevation of troponin in the first postoperative days and mortality in 30 days

Troponin T (ng / mL)	Patients n (%)	Deaths 30 days after surgery n (%)	Adjusted HR (CI 95%)
< 0.01	13,376 (88.4)	134 (1.0)	1.0
0.02	494 (3.3)	20 (4.0)	2.41 (1.33-3.77)
0.03 -0.29	1,121 (7.4)	105 (9.3)	5.00 (3.72-6.76)
≥ 0.30	142 (0.9)	24 (16.9)	10.48 (6.25-16.62)

HR: hazard ratio. Adapted from Devereaux et al.^35^

These studies also established perioperative troponin thresholds for MINS: (1) a troponin T > 0.03 ng/mL^11^ and (2) elevation in highly sensitive troponin T (hsTnT) from 0.02 to < 0.065 ng/mL with an absolute change of at least 0.005 ng/mL (which was independently associated with 30-day mortality), or hsTnT > 0.065 ng/mL.^3^ Although no study has established ideal thresholds for troponin I for MINS, there is no preference for troponin T over I. Until research establishes troponin I thresholds for MINS, clinicians must define an elevation as any value above the 99^th^ percentile upper reference limit for each troponin I assay.^6^

The elevation of troponin remains controversial in specific surgical populations, such as elderly patients with hip fractures. Some studies have reported an increase in short and long-term mortality,^37^^,^^38^ while others showed no change.^39^^,^^40^ An observational cohort study^41^ on this type of population included 312 patients and showed that the isolated increase in troponin was not predictive of death and/or readmission or any other outcome, including postoperative complications, hospitalization, intensive care unit (ICU) admission, or functional status.

Prospective studies evaluating adults undergoing non-cardiac surgery and troponin measurements after surgery demonstrate that 13% to 18% develop MINS within 30 days of surgery.^3^^,^^12^ Among these individuals who develop MINS, 22% to 29% met the criteria for the universal definition of MI.^32^ Most of the perioperative MI and MINS occur in the first 48 hours after surgery, and the perioperative MI can occur without ischemic symptoms. This disparity can be explained, in part, by the use of narcotics and sedatives after surgery.^6^

Considering that most of MINS’ cases will go unnoticed without troponin monitoring, the recommendation is to measure the biomarker levels in at-risk patients on days 1, 2 and 3 after non-cardiac surgery while the patient is in the hospital.^6^ Based on the cost-consequence analysis of the VISION study, we can define patients at risk those who are older than 65 years or with a history of atherosclerotic disease.^42^ (Figure 3)

**Figure 3:** Evaluation for patients undergoing non-cardiac surgery. Adapted from Devereaux & Szczeklik W.^6^

BNP and NT-proBNP are biomarkers released into the systemic circulation in response to the stretching of the left atrial myocardium. They are also released in response to ischemia, inflammation, and neuroendocrine stimuli. Preoperative concentrations of natriuretic peptides are strong predictors of perioperative cardiac events, including mortality, MI and heart failure.^43^ In patients undergoing vascular surgery, measurement of natriuretic peptides for preoperative risk assessment substantially improves predictions based on tools such as the RCRI.^22^ Recently, a sub-analysis of the VISION registry showed that in individuals over 45 years, NT-proBNP dosing in the preoperative period identified individuals at greatest risk for MINS and promoted incremental value on the RCRI scale by identifying individuals at greatest risk cardiovascular.^44^

Rodseth e cols.^45^ conducted a systematic review of 2,179 patients and showed that elevated preoperative BNP in concentrations higher than 92ng/L or concentrations of preoperative NT-proBNP higher than 300ng/L were strong predictors of death or non-fatal MI within 30 days after surgery (OR, 3.4 [95% CI 2.6–4.5; p < 0.001]) and 180 days or more after surgery (OR 2.6 [95% CI 2.0–3.4; p < 0.001]). A model using preoperative BNP correctly reclassified 16% more high-risk patients and 15% more low-risk patients than a model based only on preoperative baseline risk factors. Adding BNP or NT-proBNP measurements, it was observed that high preoperative values increase the predictive capacity of a composite outcome of death and non-fatal MI in 30 days (adjusted OR, 3.7 [95% CI 2, 2–6.2]) and 180 days (adjusted OR 2.2 [95% CI 1.9– 2.7]) after non-cardiac surgery.^45^

European Society of Anesthesiology guidelines for preoperative risk assessment of non-cardiac surgery recommend preoperative measurement of natriuretic peptides in high-risk patients scheduled for general or orthopedic surgery and in intermediate and high-risk patients scheduled for vascular surgery or chest surgery.^46^

The Canadian Society of Cardiology Guidelines for Noncardiac Surgery^21^ recommend measuring BNP or NT-proBNP before surgery to improve the estimate of perioperative cardiac risk in patients 65 years of age or older. In those aged 45 to 64 years with significant cardiovascular disease or an RCRI score ≥ 1. In addition, patients with high concentrations of biomarkers must have troponin measured in the first two postoperative days.^21^

The Third Guideline for Perioperative Cardiovascular Evaluation of the Brazilian Society of Cardiology recommends for those patients of intermediate or high risk, the active search for the occurrence of cardiovascular events, through monitoring in a semi-intensive unit or ICU environment. The risk of an event of ischemic nature requires electrocardiographic monitoring and measurement of myocardial injury markers (troponin) until the third day after surgery, a period in which most cardiovascular events are concentrated.^47^

### Treatment

The complexity of the mechanisms that contribute to perioperative MI makes it difficult to treat MINS. Some of the treatment options established for the non-operative scenario, such as the use of antiplatelet agents, pose a high risk due to the possibility of postoperative bleeding. In addition, there is little data from randomized clinical trials on the treatment of MINS.^16^

### Preoperative therapy for MINS prevention

A point that remains relatively unaddressed at MINS is prevention. The proposed mechanisms of MI in the perioperative period include fissures or rupture of the atherosclerosis plaque, in a condition characterized by hypotension, tachycardia, high catecholamine levels and arrhythmias . In the prevention of MINS, a balance must be struck between providing adequate antithrombotic coverage without increasing the risk of bleeding and minimizing the adverse effects of increased sympathetic impulse, avoiding hypotension.^14^

To date, perioperative patient care has largely focused on the prevention of MI, and few studies have attempted to determine the impact of secondary preventive therapies for MI in patients with isolated troponin elevation.^48^

In the past, beta-blockers were recommended in patients at vascular risk who underwent cardiac surgery of intermediate or high risk. However, after the publication of the POISE trial^49^ that evaluated the preoperative use of beta-blockers, the risk-benefit ratio was considered unfavorable. The POISE study recruited 8,351 patients and demonstrated that metoprolol significantly reduced the incidence of MI compared to placebo, but significantly increased the risk of stroke and clinically important bradycardia. In addition, the metoprolol group had a significantly higher risk of mortality compared to placebo. So, the main preoperative strategy of using beta-blockers proved to be ineffective and even dangerous for those patients who are not yet taking the drug to treat their pre-existing cardiac conditions.^49^ In patients with proven, or at high-risk of, coronary heart disease, it is appropriate that they receive beta-blockers for long-term outcome benefits, irrespective of any impending surgery.^50^

Perioperative observational data suggest that patients with MINS benefit from aspirin and statin therapy. Although the use of aspirin would aim to avoid a state of postoperative hypercoagulability that can result in ischemic events, the role of aspirin in preventing MINS remains controversial.^10^ In a substudy of 415 patients who had a perioperative MI in the POISE trial, a multivariate analysis demonstrated a lower risk of death at 30 days in patients using aspirin and statins.^6^ On the other hand, the results of the POISE 2 study showed that aspirin did not result in an improvement in the rate of cardiovascular events and mortality within 30 days, instead causing a significant increase in bleeding compared to placebo. The authors postulated that the increase in cardiovascular events may be related to increased bleeding, causing a supply and demand failure.^51^

However, the POISE 2 study^51^ has some shortcomings that potentially limit its applicability. For example, the time of aspirin administration in the preoperative period has not been standardized. Based on the inclusion criteria, aspirin would have been indicated for secondary prevention in most patients in the study. Withdrawing aspirin in these patients may have put them at increased risk for cardiac events and stroke, as withdrawal from aspirin treatment results in increased thromboxane A2 levels and decreased fibrinolysis.^52^

Observational study by Foucrier et al.^53^ included 66 patients who had troponin elevation after vascular surgery and 132 paired controls who did not have troponin elevation after surgery. The primary outcome was the occurrence of a major cardiac outcome, that is, MI, coronary revascularization, or pulmonary edema with hospitalization in one year. Intensification of cardiovascular medication was defined as the introduction or increase of the dose of at least one of the following four medications – antiplatelet agents, statins, beta-blockers, and angiotensin-converting enzyme inhibitors. Patients without intensified cardiovascular treatment had an RR of 1.77 (95% CI 1.13–2.42) for the primary outcome, compared to the matched control group. On the other hand, patients who received intensification of cardiovascular treatment had a similar risk for the primary outcome compared with the control group (RR 0.63; 95% CI 0.10–1.19).^53^ Also, a meta-analysis demonstrated that interruption of aspirin in the perioperative period in patients with or without risk of ischemic heart disease was associated with a three-time greater risk of serious adverse cardiac events.^54^

Cardiovascular complications are the most important cause of perioperative morbidity and mortality among patients undergoing vascular surgery. The protective effects related to the use of statins seem to be based on lipid-lowering and other properties of these drugs, such as improvement in endothelial function, in hemostasis and inflammation, which result in coronary plaque stabilization. Clinical trial showed that the incidence of cardiovascular events in the first six months after surgery, including death from cardiac causes, nonfatal acute MI, ischemic stroke, and unstable angina, can be reduced with perioperative use of atorvastatin in patients who must undergo vascular surgery, regardless of their serum cholesterol concentration.^55^

The Third Guideline for Perioperative Cardiovascular Evaluation of the Brazilian Society of Cardiology recommends that for patients receiving aspirin for secondary prevention, the medication should be kept at the maximum dose of 100mg per day. Meta-analysis data suggest that this relationship is favorable for the majority of patients in the perioperative period. Neurosurgeries, due to the high morbidity and mortality of related bleeding, are an indication for aspirin suspension seven days before surgery.^47^

The main guidelines recommend the continuation of statin therapy in the perioperative period, and the initiation of statins in patients undergoing vascular surgery.^21^^,^^47^^,^^56^^,^^57^ The VISION study showed that patients who took statins preoperatively had significantly lower rates of MINS and all-cause mortality. Statins should be started at least two weeks before surgery to promote the plaque-stabilizing effects and anti-inflammatory action.^14^

The latest American College of Cardiology/American Heart Association (ACC/AHA) guidelines do not recommend coronary revascularization before non-cardiac surgery.^57^ Preoperative coronary artery revascularization did not reduce long-term mortality or postoperative MI compared to drugs in a study with more than 5,000 patients undergoing vascular surgery.^58^ Myocardial revascularization surgery and percutaneous coronary intervention are procedures associated with a significant risk.^59^ Patients with coronary stents after percutaneous coronary intervention are at risk of in-stent thrombosis during surgery, especially if antiplatelet drugs are discontinued in the perioperative period. Revascularization before non-cardiac surgery is recommended only for patients in whom revascularization is indicated regardless of surgery.^57^

In the perioperative phase, to prevent cardiovascular events, the anesthesiologist must limit hypothermia and immediately treat hypotension and tachycardia, possibly avoiding beta-blockers.^48^

### Postoperative care

In the postoperative period, hypotension, tachycardia, hypoxia, bleeding, and pain promote an imbalance in the supply and consumption of oxygen in the myocardium, increasing the risk of injury, and must be detected and corrected early.^48^

In the management of MINS, the use of aspirin and statin may be recommended in patients who do not meet the criteria for acute coronary syndrome, as this has been shown to improve results.^21^ Theoretically, beta-blockers may have a role in the management of the imbalance between oxygen supply and demand that results in MINS. However, the benefits of using beta-blockers and angiotensin-blocking agents should be weighed against the risk of hypotension, which can accentuate myocardial damage.^14^ Additional studies are needed to provide more robust evidence on the use of these drugs. Patients who meet the criteria for acute coronary syndrome should be treated according to current clinical practice guidelines.^14^ Invasive procedures to investigate obstructive coronary disease in patients with MINS would not be indicated, except for patients who present criteria for STEMI or patients with hemodynamic or electrical instability.

The MANAGE study examined the use of dabigatran for the treatment of MINS. The authors found that subjects in the intervention arm had lower rates of major vascular events (n = 97; 11% - patients assigned to dabigatran vs 133 – 15% – in placebo group, p = 0.0115), with similar bleeding complications compared to the placebo arm (n = 29 patients; 3% - dabigatran group vs. n = 31; 4% – placebo group, p = 0.76).^7^ However, the study has some limitations in its design and results. The study ended early, the primary outcome was modified in the middle of the study, and drug discontinuation rates were high. Despite its deficiencies, the study paves the way for further research in this area.^14^

## Conclusions

MINS represents a new clinical entity characterized today as an emergent health problem in view of the growing number of non-cardiac surgeries and their high prevalence. MINS is a marker of future vascular events and its early detection with the use of biomarkers should be performed in all medium or large surgeries in individuals aged 65 years or over and in those with previous atherosclerotic disease regardless of age. Multicenter registries and clinical trials have been progressively developed with the aid of perioperative medicine aiming at improving cardiovascular surveillance and treatment of this group of patients.
